# A Dynamical, Radically Embodied, and Ecological Theory of Rhythm Development

**DOI:** 10.3389/fpsyg.2022.653696

**Published:** 2022-02-24

**Authors:** Parker Tichko, Ji Chul Kim, Edward W. Large

**Affiliations:** ^1^Department of Music, Northeastern University, Boston, MA, United States; ^2^Perception, Action, Cognition (PAC) Division, Department of Psychological Sciences, University of Connecticut, Mansfield, CT, United States; ^3^Center for the Ecological Study of Perception and Action (CESPA), Department of Psychological Sciences, University of Connecticut, Mansfield, CT, United States; ^4^Department of Physics, University of Connecticut, Mansfield, CT, United States

**Keywords:** neural resonance theory, musical rhythm development, neural oscillations and entrainment, music enculturation, dynamical systems theory, ecological psychology

## Abstract

Musical rhythm abilities—the perception of and coordinated action to the rhythmic structure of music—undergo remarkable change over human development. In the current paper, we introduce a theoretical framework for modeling the development of musical rhythm. The framework, based on Neural Resonance Theory (NRT), explains rhythm development in terms of *resonance* and *attunement*, which are formalized using a general theory that includes non-linear resonance and Hebbian plasticity. First, we review the developmental literature on musical rhythm, highlighting several developmental processes related to rhythm perception and action. Next, we offer an exposition of Neural Resonance Theory and argue that elements of the theory are consistent with dynamical, radically embodied (i.e., non-representational) and ecological approaches to cognition and development. We then discuss how dynamical models, implemented as self-organizing networks of neural oscillations with Hebbian plasticity, predict key features of music development. We conclude by illustrating how the notions of dynamical embodiment, resonance, and attunement provide a conceptual language for characterizing musical rhythm development, and, when formalized in physiologically informed dynamical models, provide a theoretical framework for generating testable empirical predictions about musical rhythm development, such as the kinds of native and non-native rhythmic structures infants and children can learn, steady-state evoked potentials to native and non-native musical rhythms, and the effects of short-term (e.g., infant bouncing, infant music classes), long-term (e.g., perceptual narrowing to musical rhythm), and very-long term (e.g., music enculturation, musical training) learning on music perception-action.

## Introduction

Musical rhythm abilities involve the perception of and coordinated action to the rhythmic structure of music—structures that vary in their complexity across cultures. How do humans develop the ability to coordinate perception-action to musical rhythms? How do infants and children acquire knowledge about culture-specific rhythmic structures? What mechanisms and processes might explain developmental changes in musical rhythm abilities over the lifespan? Are there general dynamical principles that can explain the development of musical rhythm across different scales of organization (e.g., neural, behavioral, social) in the organism-environment system? Over the last several decades, research on musical rhythm development has illuminated several features regarding the ontogenetic origins of musical rhythm: prenatal learning, postnatal developmental plasticity of musical rhythm, biases for and constraints on musical rhythm perception and action, the effects of social context on rhythm abilities, and continued lifespan development. Despite this growing characterization of typical developmental trajectories, little research has investigated developmental *mechanisms* or processes that might explain these features of musical rhythm development. In the current paper, we introduce a theoretical framework for modeling key aspects of rhythm development that makes explicit claims about the developmental mechanisms underlying rhythm ontogeny. The framework, based on Neural Resonance Theory (NRT), explains rhythm development in terms of *resonance* and *attunement*. These constructs are formalized using a general theory of non-linear resonance that is implemented in self-organizing networks of neural oscillations to explain developmental plasticity and biases for rhythm perception and action. We discuss how these oscillatory neural networks can be employed to model developmental changes in musical rhythm abilities and highlight some recent implementations of NRT that have modeled key aspects of music development. Finally, we conclude by suggesting that the NRT framework can be used to generate and test empirical predictions on rhythm development, such as the kinds of native and non-native musical rhythmic structures infants and children can learn, steady-state evoked potentials to native and non-native musical rhythms, and the effects of short-term (e.g., infant bouncing, infant music classes) and long-term learning (e.g., music enculturation, musical training) on music perception-action.

## The Structure of Musical Rhythm

Music is a high-level cognitive capacity that exists universally across human cultures. Similar to the structural underpinnings of language (e.g., linguistic syntax), music has an abstract, rhythmic-harmonic structure that is thought to convey socio-affective meaning (Lerdahl and Jackendoff, 1983; Patel, 2010). At a fundamental level of organization, musical events (e.g., notes, chords) are arranged, accented, and sustained across time through the organizing principles of rhythm and meter. Musical rhythm refers to patterns of stress and timing of individual acoustic events, while musical meter reflects the organization of musical events on multiple, hierarchically nested timescales. At the principal level in the hierarchical organization, music has a basic beat, the *tactus*, often described as an underlying pulse of a musical work (Cooper and Meyer, 1963; Lerdahl and Jackendoff, 1983; London, 2004; Large et al., 2015). The beat, while not necessarily the slowest or fastest rhythmic component of a musical work, is often the most perceptually salient level of metrical organization—the level at which listeners and dancers behaviorally entrain to music, such as tapping their feet or nodding their heads. Additionally, musical meter reflects alternating patterns of strong-and-weak beats, such that some beats are physically or perceptually accented relative to other (Cooper and Meyer, 1963; Lerdahl and Jackendoff, 1983; London, 2004; Large et al., 2015). Finally, music also contains faster events (London, 2004; Large et al., 2015), collectively called “rhythmic patterns” or “rhythmic groups,” that reflect the relative durations between auditory events, and, importantly, the perceptual grouping of these events (Cooper and Meyer, 1963).

While rhythm and meter are prominent, if not universal structures of music, these structures do vary across cultures in systematic ways. In Western music, beats are commonly grouped into units of twos, threes, or fours, forming duple, triple, or quadruple meters, respectively. For instance, a waltz has a triple-metered beat pattern of strong-weak-weak. This patterning creates a robust 1-2-3 feel, with emphasis felt on beat position 1 in the larger three-beat grouping. Strong beat positions are called the “down beat,” such as beat position 1 in a waltz, while weak beat positions are called the “off beat,” such as beat positions 2 and 3 in a waltz. Further, Western music is typically characterized by even and periodic temporal relationships between strong beats which are usually denoted by simple integer ratios (e.g., 1:1 or 2:1). In the time domain, this often reflects evenly spaced accented beats called isochronous meters (e.g., a 500-ms temporal interval between beat 1 and beat 2, a 500-ms temporal interval beat 2 and beat 3, and so on; exhibiting a 1:1 integer ratio between beats at the same metrical level). Non-Western music, however, particularly the music of Eastern Europe and the Balkan regions, often contains metrical structures in which beats, usually at the tactus level, are unevenly spaced, referred to as non-isochronous meters (London, 2004). These metrical structures include relatively more complex ratios between beats, such as 3:2 (e.g., a 750-ms temporal interval between beat 1 and beat 2, a 500-ms temporal interval between beat 2 and beat 3, exhibiting a 3:2 ratio between beats) (London, 2004; Ullal-Gupta et al., 2014).

## Developmental Changes in Musical Rhythm

Over the course of ontogeny, the perception and action of musical rhythm undergo remarkable developmental change (Ullal-Gupta et al., 2013; Provasi et al., 2014; Hannon et al., 2018). Beginning with the maturation of the cochlea and the central auditory system (Lahav and Skoe, 2014), the human fetus begins to respond to prominent auditory-rhythmic structures originating from the extra-uterine environment, such as the prosodic information of speech and music (Ullal-Gupta et al., 2013). Both physiological and behavioral recordings taken of the fetus and newborn indicate that learning the structure of exogenous auditory-rhythmic inputs likely starts *in utero*, particularly during the final trimester of prenatal development (DeCasper and Fifer, 1980; DeCasper and Spence, 1986; Fifer and Moon, 1994; Sansavini, 1997; Giovanelli et al., 1999; James et al., 2002; Kisilevsky et al., 2003, 2004; Granier-Deferre et al., 2011b). Changes in fetal heart rate and movement patterns, for instance, suggest that fetuses already are sensitive to the rhythmic structure of exogenous auditory stimuli (Granier-Deferre et al., 2011b), even recognizing familiar passages of music heard in the womb (Hepper, 1991; Kisilevsky et al., 2004). This initial sensitivity to and recognition of familiar rhythmic patterns, during prenatal development, has been found to persist into postnatal development: newborns respond to and recognize familiar musical themes initially heard *in utero* for days and several weeks into postnatal life, suggesting that human fetuses and newborns retain information about the rhythmic structure of external auditory inputs at least over a short time span (Hepper, 1991; Granier-Deferre et al., 2011a).

The results from these studies strongly suggest that experiences related to ontogeny of musical rhythm occur even prior to birth. In addition to prenatal experience with exogenous rhythms, musical rhythm development may also involve non-obvious forms of rhythmic stimulation. For instance, several theorists has proposed that experience with rhythmic sounds endogenous to the intrauterine environment (e.g., the maternal heart beat) (Ullal-Gupta et al., 2013; Teie, 2016; Parncutt and Chuckrow, 2019) and vestibular stimulation arising from maternal movements (Provasi et al., 2014; Rocha et al., 2020) may influence early developmental trajectories of rhythmic abilities. Indeed, many intrauterine biological sounds (Murooka et al., 1976; Busnel, 1979), such as maternal cardio-vascular and respiratory sounds, are inherently rhythmic. The maternal heartbeat, for instance, is a prominent rhythmic stimulus in the womb (Querleu et al., 1988) that could provide the fetus with its first sense of pulse (Ullal-Gupta et al., 2013; Teie, 2016). In support of these theories, accumulating evidence indicates that prenatal experience with endogenous rhythmic sounds promotes typical neural and behavioral development of the fetus and newborn (DeCasper and Sigafoos, 1983; Doheny et al., 2012; Lahav and Skoe, 2014; Webb et al., 2015), including the development of auditory cortex (Webb et al., 2015). Prenatal experience with non-obvious forms of rhythmic stimulation, thus, may be important for the development of neural and physiological systems which support later rhythm development. However, to our knowledge no study has empirically linked prenatal experience with endogenous rhythmic sounds to aspects of rhythm development.

Thus, findings regarding auditory-rhythm learning during fetal development suggest that rhythmic abilities which emerge at or near birth may reflect prenatal learning mechanisms that operate over extra-uterine auditory-rhythmic inputs (e.g., music, speech), and, possibly, non-obvious forms of rhythmic stimulation (e.g., maternal heartbeat, vestibular stimulation from maternal movement). Even at early stages of postnatal development, newborn and young infants already display a remarkable range of rhythmic abilities. For example, some evidence suggests that newborns actively anticipate and predict the beat of musical rhythms (Winkler et al., 2009). Using an event-related potential (ERP) paradigm, Winkler et al. (2009) measured newborn infants’ brain responses [e.g., the mis-match negativity (MNN) response] to rock drum beats with an omitted rhythmic event that either occurred on a theoretically weak beat position (e.g., beat position 2, the “off beat”), or theoretically strong beat position (e.g., beat position 1, the “down beat”). The MMN evoked by the omission of the strong beat position was found to have a larger response amplitude in the MMN, suggesting that human newborns actively anticipate musical beats. Further, newborns, at 2 months of age, and young infants, at 5 months of age, can discriminate between contrasting auditory rhythms (Chang and Trehub, 1977; Demany et al., 1977) and detect temporal disruptions to auditory rhythms (Otte et al., 2013), indicating that basic, perceptual mechanisms of rhythmic grouping have already developed by early infancy. Older infants, at 6 and 12 months, also discriminate contrasting auditory rhythms, though there may be age-related changes in the kinds of cues (e.g., absolute and relative timing cues) that younger and older infants use to do so (Morrongiello, 1984).

Despite the ability of younger and older infants to perceptually group auditory events into rhythmic patterns, they rarely produce synchronized movements to musical rhythm. Rather, the ability to behaviorally entrain to musical rhythm, an ability called sensorimotor synchronization (SMS) (Repp, 2005; Repp and Su, 2013), follows an extended developmental trajectory across infancy, childhood, adolescence, and adulthood (Drake et al., 2000; Eerola et al., 2006; McAuley et al., 2006; Kirschner and Tomasello, 2009, 2010; Zentner and Eerola, 2010; Kirschner and Ilari, 2014; Ilari, 2015; Yu and Myowa, 2021). A few studies have attempted to assess SMS during infancy (Zentner and Eerola, 2010; Fujii et al., 2014; Ilari, 2015), but only one study has found that, in rare cases, young infants will spontaneously move and synchronize their movements to the rhythmic structure of music (Fujii et al., 2014). In a sample of 3- and 4-month-old infants, Fujii et al. (2014) reported two infants who were able to synchronize leg and arm movements to the rhythmic structure of musical stimuli. On average, however, the young infants in this study did not move more frequently or synchronize to music, relative to a control condition of silence. Unlike young infants, older infants (Zentner and Eerola, 2010; Ilari, 2015; Rocha and Mareschal, 2017), beginning around 5 months, do spontaneously move to music and flexibly adjust their rate of movement to track changes in musical tempo. Zentner and Eerola (2010) investigated developmental changes in infants’ and toddler’s spontaneous movement to recordings of natural music, rhythm percussion stimuli (e.g., programmed, isochronous drum beats), and natural speech. Infants and toddlers from 5- to 24-months, sampled in a cross-sectional design, all moved more rhythmically to naturalistic music and percussive stimuli relative to speech stimuli. Moreover, infants, in response to music, modulated their bodily movement to mirror tempo fluctuations that occurred in the natural music stimuli (i.e., increasing or decreasing bodily movements when presented with musical tempo changes). However, these movements were not synchronized to the auditory stimuli. Another study conducted with a sample of 2- to 4-year-old children reported similar findings (Eerola et al., 2006). Using a similar paradigm, children were encouraged to move to music naturally. Similar to the infants’ performance, children’s rhythmic movements to music were, in general, not synchronized to the beat of the musical stimuli. Moreover, unlike infants’ adjustment in rhythmic movements to tempo changes, children’s movements did not adjust to tempo changes (Eerola et al., 2006).

Beyond infancy, sensitivity to rhythmic structure continues to develop during older childhood, adolescence, and late adulthood (Wilson et al., 1997; Thompson et al., 2015; Einarson and Trainor, 2016; Nave-Blodgett et al., 2020). For instance, at both 7 and 9 years of age, children can categorize rhythms as either adhering to a metrical structure or not (Wilson et al., 1997). However, Wilson et al. (1997) reported that this classification ability was stronger in a sample of 9-year-olds relative to 7-year-olds, suggesting there may be age-related changes in children’s perception of musical meter. Moreover, when asked to discriminate between rhythms within a metrical category (e.g., metrical rhythm vs. metrical rhythm, non-metrical rhythm vs. non-metrical rhythm), children’s performance was much worse than discriminating across metrical categories (Wilson et al., 1997), indicating that rhythm and meter development may reflect different ontogenetic trajectories. Consistent with this, Nave-Blodgett et al. (2020) found that adults, but not children or adolescents, were able to simultaneously track two distinct metrical levels of musical stimuli (e.g., both beat and meter levels). Further, while young children exhibited some sensitivity to the beat level, they were not sensitive to the measure level in music, like adults. Together, these studies indicate that rhythm and meter perception continue to develop through adulthood.

Similarly, the development of SMS also undergoes age-related changes during childhood and adulthood. Around the age of five and six, children begin to produce overt, synchronized movements to exogenous rhythms, such as music (Drake et al., 2000; Volman and Geuze, 2000; McAuley et al., 2006; Thompson et al., 2015; Nave-Blodgett et al., 2020). This behavior, however, is not ubiquitous across all rhythmic stimuli. In a cross-sectional study, Drake et al. (2000) investigated the ability of children, aged 4 to 10 years, to synchronize to a musical recording of Ravel’s Bolero, isochronous auditory beats, and auditory rhythmic stimuli. Interestingly, children, at all ages, were found to synchronize to Ravel’s Bolero (i.e., a ceiling effect across all ages). However, synchronization to isochronous beats and rhythmic stimuli increased as a function of developmental age (Drake et al., 2000). Another study found that the ability to flexibly adapt SMS across a range of rhythmic timescales follows a non-linear trend over the lifespan: McAuley et al. (2006) calculated a measure of entrainment flexibility in cross-sectional samples of human participants that spanned the human lifespan from childhood to late adulthood and found that the measure followed a quadratic trend, indicating that the range of entrainment is initially narrow, widens throughout mid-life, and then narrows near the end of the lifespan (McAuley et al., 2006).

In addition to age-related changes in synchronization to musical rhythm, cultural and socialization contexts are also believed to modulate rhythm abilities (Kirschner and Tomasello, 2010; Kirschner and Ilari, 2014; Yu and Myowa, 2021). For instance, Kirschner and Ilari (2014) investigated whether children’s synchronization to musical rhythms in various social contexts (e.g., with an experimenter visible, an experimenter hidden, or in solo) would influence subsequent prosocial behavior. While the authors predicted that entrainment in more interactive social contexts, such as drumming along with a visible experimenter, would engender pro-social behavior, the researchers found no effect of social context. Interestingly, however, cross-cultural differences in rhythmic synchronization emerged: In samples of German and Brazilian 3-year-old children, Brazilian children synchronized spontaneously to a musical drum better than German children. In follow-up parental interviews, the children in the Brazilian sample had a greater history of musical activities relative to the German children, indicating a possible effect of culture on rhythm synchronization. Relatedly, several studies have demonstrated that infants’ and young children’s rhythmic behavior is influenced by the presence of a social partner: for instance, older infants are more likely to adjust the tempo of their movements (Rocha and Mareschal, 2017) and young children are more likely to synchronize to the beat of music (Kirschner and Tomasello, 2010; Kirschner and Ilari, 2014), in the presence of another social partner. Rhythm development, thus, may be influenced by cultural and socialization processes (Kirschner and Ilari, 2014).

Similar to the longitudinal development of SMS across the lifespan, an individual’s preferred intrinsic tempo, operationally defined as the spontaneous motor tempo (SMT) (i.e., the rate of tapping in the absence of a reinforcing, external rhythm) or the preferred perceptual tempo (PPT) (i.e., preference for stimulus presentation rates), also undergoes drastic change over the lifespan (Bobin-Bègue and Provasi, 2005; McAuley et al., 2006; Rocha et al., 2020). Given the fine-grained sensorimotor demands of SMT tasks, such as tapping experiments, research has generally studied age-related changes in preferred tempo beginning with childhood, overlooking infancy. [Though, see recent work from Rocha et al. (2020)]. In a large sample spanning the human lifespan (age 4–75+ years), McAuley et al. (2006) found that intrinsic tempo, as measured by SMT and PPT, declines across childhood, adulthood, and late adulthood: For instance, young children (4–7 years of age) were found to have an SMT at ∼300 ms (∼3.3 Hz), while older children exhibited an SMT around ∼520 ms (∼2 Hz). Further, adults preferred a spontaneous tapping rate at ∼630 ms (∼1.6 Hz), while the older adults preferred to spontaneously tap at ∼650 ms (∼1.5 Hz). Collectively, these data indicate preferred tempo decreases in frequency, beginning in early childhood and continuing through late adulthood.

### Developmental Plasticity of Musical Rhythm

As rhythm abilities begin to emerge during development, they become functionally specialized, likely as a consequence of experience-dependent plasticity (Hannon and Trehub, 2005a,b; Tichko and Large, 2019). Developmental plasticity of musical rhythm has been observed over multiple ontogenetic timescales, including seconds and minutes (Phillips-Silver and Trainor, 2005, 2007), weeks and months (Hannon and Trehub, 2005a,b; Gerry et al., 2010; Zhao and Kuhl, 2016), and years (Smith, 1983; Upitis, 1987; Smith and Cuddy, 1989; Drake, 1993; Slater et al., 2013; Doelling and Poeppel, 2015; Thompson et al., 2015; Cirelli et al., 2016; Scheurich et al., 2018, 2020; Harding et al., 2019). For example, on the timescales of seconds and minutes, infants’ perception of accented beats in musical rhythm re-organizes to vestibular input that arises from bodily movement to music. In one study, Phillips-Silver and Trainor (2005) found that, after a period of bouncing infants to an unaccented musical rhythm, infants preferred listening to accented rhythms that matched the rate of their bouncing. On the timescale of months, infants’ perceptual systems gradually become fine-tuned to their musical environment over the first postnatal year of life (Lynch et al., 1990; Lynch and Eilers, 1992), resulting in culture-specific biases for music perception (Hannon and Trehub, 2005a,b; Soley and Hannon, 2010; Tichko and Large, 2019). In a pair of studies on musical rhythm perception, Hannon and Trehub (2005a,b) demonstrated that Western infants at 6 months could detect temporal disruptions to both native and non-native rhythmic structures equally well. However, by 12 months, Western infants could only detect the disruptions in music that contained beat structures native to their culture. These findings suggest that rhythm perception undergoes a gradual process of fine-tuning to culture-specific rhythmic structures over the first year of infancy. In another study, Hannon and Trehub (2005a) found that when 12-month-old Western infants were exposed to non-native music for 2 weeks, they improved on detecting disruptions in non-native beat structures. Western adults, however, did not. Thus, even as infants’ perceptual systems begin to tune to culture-specific musical rhythms, they remain highly plastic and can reorganize to novel rhythmic inputs.

Studies on active and formally structured infant music classes also illustrate the plasticity of rhythm abilities over moderate developmental timescales, such as weeks and months (Gerry et al., 2010; Cirelli et al., 2016; Zhao and Kuhl, 2016). In one study, Zhao and Kuhl (2016) randomly assigned 9-month-old infants to participate in infant music classes or a control play class for 1 month. Infants in the music class were exposed to different rhythmic structures, notably music with triple meters. Infants in the control class engaged in play, but without music. After the intervention, magnetoencephalography (MEG) was used to examine infant brain responses to rhythmic stimuli that violated a triple meter: infants who participated in the music intervention exhibited larger brain responses to the violation (i.e., larger mis-match negativity responses), relative to the infants in the play condition (Zhao and Kuhl, 2016). Similarly, another study found that neural activity in response to a metrically ambiguous rhythm was pronounced for 7-month-old infants that had a history of infant-caregiver music classes (Cirelli et al., 2016), suggesting that early music activity might modulate neural synchrony to the rhythmic structure of music. [However, when 15-month-old infants were tested after being randomly assigned to participate in music classes or no classes for 20 weeks beginning at 9- or 10-months of age, no enhancement to neural synchrony was found (Cirelli et al., 2016)].

Over the timescale of years, both passive (e.g., music listening) and active (e.g., musical training) forms of musical experience are associated with the development of musical rhythm abilities. For instance, older children and adults exposed to non-native rhythms for several weeks exhibit a lesser degree of plasticity to these non-native rhythms, relative to younger children, presumably older children and adults are more strongly enculturated to their native musical systems (Hannon et al., 2012). Music training has also been linked to rhythm abilities, particularly in older childhood and adulthood. One study found that children who participated in a year-long music program displayed superior beat synchronization abilities relative to children who did not (Slater et al., 2013). Relatedly, a body of work has found associations between musical training and enhanced rhythm perception, synchronization, and neural responses to auditory rhythms in childhood and adulthood (Smith, 1983; Upitis, 1987; Smith and Cuddy, 1989; Drake, 1993; Slater et al., 2013; Doelling and Poeppel, 2015; Thompson et al., 2015; Cirelli et al., 2016; Scheurich et al., 2018, 2020; Tichko and Skoe, 2018; Harding et al., 2019).

### Biases for and Constraints on Musical Rhythm

Despite the evidence that rhythm perception and action exhibit a remarkable degree of plasticity over multiple ontogenetic timescales, rhythm abilities are also biased toward and constrained to favor relatively simple rhythmic structures (Povel, 1981; Fraisse, 1982). Evidence regarding such biases for and constraints on musical rhythm comes from studies investigating perception-action abilities for rhythmic structures that vary in their structural complexity. For instance, some evidence suggests that older children and adults have advantages for perceiving, tracking, and reproducing musical rhythms with simple structures (Povel, 1981; Smith and Cuddy, 1989; Drake, 1993; Wu et al., 2013), such as duple meter (2:1 ratio), relative to more complex meters (Collier and Wright, 1995; Ullal-Gupta et al., 2014; Einarson and Trainor, 2016), such as ternary meter (3:1 ratio) [Though, see Drake (1997)]. These findings point to possible perception-action biases of and constraints for musical rhythm that are related to the complexity of rhythmic structure. However, such biases for relatively simple rhythmic structures (e.g., 2:1 vs. 3:1) identified in older childhood and adulthood could also reflect learning and enculturation processes involving culture-specific rhythmic structures (Ullal-Gupta et al., 2014; Zhao and Kuhl, 2016).

Interestingly, however, advantages for simple rhythmic structures have also been observed in infancy, even prior to years of exposure to culture-specific musical systems. Similar to findings with older children and adults, Bergeson and Trehub (2006) found that 9-month-old infants were better at discerning temporal changes in musical stimuli that followed a simpler duple meter, relative to a more complex triple meter. In another study, Trehub and Hannon (2009) reported that 6-month-old infants were better at detecting pitch and rhythm violations in music stimuli that followed a simpler, conventional meter (e.g., 3/4 time signature) relative to a more complex, unconventional meter (e.g., 13/8 time signature). In addition to these perceptual advantages for simpler rhythmic structures, some work suggests that there are perceptual constraints on perceiving highly complex rhythmic structures that are rarely found among the world’s music in infancy. For instance, Hannon et al. (2011) found that 5-month-old infants could detect temporal disruptions in simple (e.g., 2:1 meter) and complex (e.g., 3:2 meter) rhythms, but not highly complex (e.g., 7:4 meter) rhythms. Moreover, infants prefer to listen to music with simple rhythmic structures relative to highly complex: one study assessed 5-month-old Western and Balkan infants’ listening preferences for different rhythmic structures commonly found in Western and Balkan music relative to complex rhythmic structures rarely found in either Western or Balkan music. Infants raised in Western and Balkan cultures preferred listening to the rhythmic structures found in their respective cultures, but neither group preferred listening to the highly complex rhythms less common to the world’s music (Soley and Hannon, 2010). These findings suggest that, even during the early stages of ontogeny, there may be perceptual biases and constraints for metrical structures that reflect relatively simple integer-ratio relationships.

### Neurobiology of Rhythm Perception and Action

In addition to characterizing typical developmental trajectories for musical rhythm, previous research has identified key neurobiological mechanisms of and neural networks for rhythm perception and action. In particular, there is now mounting evidence that the nervous system, across multiple timescales, resonates to the hierarchical structure of musical rhythm and meter across auditory and motor-planning neural systems (Merchant and Honing, 2013; Patel and Iversen, 2014; Todd and Lee, 2015; Proksch et al., 2020; Cannon and Patel, 2021). Functional neuroimaging research has revealed that meter and beat perception actively recruits both auditory brain regions, such as auditory cortex, and motor-planning and motor-control brain regions, such as premotor cortex, the supplementary motor area, the putamen, and the striatum, even in the absence of overt movement to music (Bengtsson et al., 2009; Chen et al., 2008; Grahn and Brett, 2007; Grahn and Rowe, 2009; Kung et al., 2013; Gordon et al., 2018). Further, electrophysiological (EEG) and magnetoencephalographical (MEG) studies indicate that neural oscillations across auditory and motor-planning networks anticipate and resonate to the metrical structure of music (Nozaradan et al., 2011, 2012; Fujioka et al., 2015; Cirelli et al., 2016; Tal et al., 2017; Large et al., 2018). For instance, induced Beta (13–30 Hz) oscillations from auditory and sensorimotor cortices anticipate the temporal positions of musical beats (Fujioka et al., 2009, 2012, 2015), while lower frequency brain activity in the Delta (0.5–4 Hz) and Theta (4–8 Hz) ranges resonates to musical rhythms, as evinced by strong brain responses at rhythmic frequencies (Nozaradan et al., 2011; Doelling and Poeppel, 2015; Cirelli et al., 2016). In addition to reflecting rhythmic frequencies, such resonant brain responses also capture higher order features of rhythmic structure, such as imagined metrical accents that are imposed upon a metrically ambiguous musical rhythm by a listener (i.e., top-down influences) (Nozaradan et al., 2012). Collectively, these findings suggest that meter and beat perception may emerge from bi-directional, resonant interactions between auditory and motor-planning neural networks.

### Key Features of Rhythm Development

As reviewed, ontogenetic and cross-cultural investigations of musical rhythm have identified several key components of rhythm development. First, musical rhythm abilities are plastic and adapt to culture-specific rhythmic structures over multiple timescales (e.g., from seconds to years), likely beginning in the prenatal period. Second, musical rhythm perception and action are biased toward and constrained to rhythmic structures characterized by simple integer-ratio relationships, as opposed to relatively more complex integer-ratio relationships. Third, musical rhythm abilities are modulated by the social and cultural context they are embedded in, such as the presence of a social partner and other cultural conventions for music-making. And, finally, the development of musical rhythm and timing is a longitudinal process that reflects dynamic changes across the lifespan, beginning with infancy and continuing through late adulthood.

Motivated by the above findings on the development and neurobiology of musical rhythm, we propose a novel framework, based on Neural Resonance Theory (NRT), for modeling the ontogenetic origins of musical rhythm abilities. In particular, our theoretical framework explains key features of musical rhythm development, such as the developmental plasticity of, biases for, constraints on, and lifespan changes of musical rhythm, in terms of the *resonance* and *attunement* of coupled, bio-physical oscillators that span the organism-environment system. In the section below, we provide an exposition of NRT, briefly contrasting the theory with classic information-processing accounts of musical rhythm and development. Then, we relate NRT to broader movements in cognitive science, particularly ecological (Gibson, 1966), dynamical (e.g., Haken et al., 1985; Treffner and Turvey, 1993; Scott Kelso, 1995), and radically embodied (Thompson and Varela, 2001; Chemero, 2013; Dotov, 2014) approaches to cognition. Finally, we conclude by proposing a developmental model of musical rhythm based on NRT and discuss prediction about rhythm development.

### Neural Resonance Theory

Neural Resonance Theory (NRT) is a theoretical framework for understanding how the endogenous and exogenous rhythms of the brain-body-environment system self-organize to enable perception, action, and attention in a moment-by-moment manner (Large and Snyder, 2009; Large et al., 2015). In contrast to information-processing theories (Lerdahl and Jackendoff, 1983), NRT predicts that structure in rhythm perception-action arises lawfully from interactions between coupled non-linear oscillatory systems, not from symbolic representations or grammatical rules that compute rhythmic and metrical structure (Large and Kolen, 1994). In this framework, neural oscillations are not considered “representations” of exogenous rhythms, as neural rhythms are, themselves, *physical* rhythms. Oscillations, including neural oscillations, are *embodied* rhythms, and, as such, adhere to physical laws. The objective of NRT, then, is to articulate the physical laws of embodied rhythmic interactions and explain how such laws lead to the emergence of complex rhythms in both behavior and in the brain. In this regard, NRT conceives of a rhythmic “beat” as the result of lawful interactions between bio-physical oscillations that entrain to acoustic onsets in musical and speech inputs. The psychological experience of musical rhythm is explained by NRT as an *emergent rhythm* that arises, at the psychological level, from multiple non-linear oscillations distributed across the organism (e.g., neurobiological rhythms, rhythmic patterns of action) and the environment (e.g., the rhythmic structure of speech and music).

In the tradition of dynamical systems approaches to perception, action, and development (Haken et al., 1985; Scott Kelso, 1995; Thelen and Smith, 1996; Warren, 2006), NRT models musical rhythm cognition and its development using the mathematical language of non-linear dynamical systems—in particular, dynamical systems models of oscillation. While there is both a rich and recent history in the behavioral and neural sciences of using dynamical systems models of oscillation to explain rhythmic phenomena (Haken et al., 1985, 1996; Schmidt et al., 1990; Large and Jones, 1999; Frank et al., 2000; Wilson and Wilson, 2005; Doelling et al., 2019), our proposed model of musical rhythm perception and action is based on the canonical model framework for weakly connected neural networks (Hoppensteadt and Izhikevich, 1996a,b), which we extend to encompass networks of oscillators with multiple frequencies (Large et al., 2010) and Hebbian plasticity (Tichko and Large, 2019; Kim and Large, 2021; Tichko et al., 2021). The canonical model is a generic mathematical model of oscillatory networks derived from a model of the underlying physiology (Wilson and Cowan, 1972). It assumes that oscillations arise from local interactions of excitatory and inhibitory neural subpopulations (e.g., pyramidal cells and interneurons) and that the coupling between two oscillators is determined by synaptic connections between two excitatory and two inhibitory subpopulations (Hoppensteadt and Izhikevich, 1996a). The derivation produces a dimensionless, scale-free dynamical system (Hoppensteadt and Izhikevich, 1996a) that can be analyzed and simulated to make general predictions (Kim and Large, 2015, 2019, 2021) about musical rhythm cognition and its development (Velasco and Large, 2011; Large et al., 2015; Tichko and Large, 2019; Tichko et al., 2021).

The mathematical models of NRT are derived from biophysical models of nervous system dynamics (Hoppensteadt and Izhikevich, 1996a,b; Large et al., 2010), but are formulated and motivated specifically at the psychological level, to produce a physics of perception and action at the ecological scale. This positioning of NRT, both at the psychological level and the ecological scale, allows the theory to explain and predict how individuals perceive and act to complex rhythmic patterns—for instance, how individuals perceive and coordinate their behaviors to music. Moreover, this approach affords two distinct advantages in the study of rhythm perception-action and its development: first, the dynamical systems approach leads to laws of structure, which generate strong, falsifiable predictions at the behavioral level. Secondly, our mathematical framework, while motivated at the psychological level, connects naturally with models and concepts at the neurophysiological level—neurons and neural networks, themselves, are also non-linear dynamical systems. Thus, as the fundamental unit of analysis is an emergent rhythm, it is natural to attempt to observe the emergent rhythms posited by NRT as a *process* that is directly embodied in dynamic, oscillatory neural activity that can be measured using electrophysiological and neuro-imaging techniques (e.g., EEG, MEG, local-field potentials).

The aim of NRT, then, is to elucidate physical principles of perceiving-acting systems, particularly those principles which govern rhythm perception and action. Such an approach shares many similarities with ecological (Gibson, 1966; Michaels and Carello, 1981), dynamical systems (Scott Kelso, 1995), synergetics (Turvey, 2007), and radical embodied (Chemero, 2013) movements in cognitive science. While there are important distinctions across these approaches (see Dotov, 2014 for an overview), a common theme is advancing a non-representational approach (i.e., a “radically embodied” approach) to cognition and cognitive development (Chemero, 2013; Dotov, 2014). In particular, we believe that the physical principles of *resonance* and *attunement*, those articulated by NRT, share many similarities with concepts from the non-representational, ecological psychology of Gibson (1966). Writing on the nervous system, J. J. Gibson rejected a computational interpretation of neural function, propounding, instead that the nervous system *resonates* to ecological information in energy flows from the environment. Moreover, Gibson argued that such resonance depends upon the organism’s *attunement* to the environment (i.e., how the organism is coupled to the environment), likening organismal *resonance* and *attunement* to the self-tuning of a radio to detect radio waves in the ambient environment (Gibson, 1966; Michaels and Carello, 1981; Raja, 2020, 2019).

Conceptually, NRT has two fundamental components related to Gibson’s notions of resonance and attunement (Gibson, 1966):

1. Resonance^1^ : Under NRT, structural regularities in perception, action, and attention emerge from the dynamics of bio-physical resonance. The theory predicts law-governed interactions between the (a) stimulus and bio-physical oscillations and (b) between bio-physical oscillations of various frequencies.
2. Attunement: Under NRT, organisms attune to structural regularities in the environment by learning connections strength and relative phase relationships between physical and biological oscillations. This process occurs on multiple, hierarchically nested timescales, accounting for the development of rhythmic perception and action over the lifespan of the organism.

While Gibson employed the notions of resonance and attunement metaphorically, within NRT, Gibsonian resonance is construed formally as the non-linear, resonant properties of bio-physical oscillations that resonate to exogenous inputs and activity from other coupled bio-physical oscillators. Considered in this way, and similar to Gibson’s claim, the nervous system does not *compute* the properties of music, but, rather, *resonates* to musical patterns over multiple spatio-temporal scales in a law-governed manner (Large, 2010). Further, Gibson’s notion of attunement can be considered under NRT as a process of Hebbian plasticity that changes the couplings across the brain-body-environment system. Hebbian plasticity, over the course of ontogeny, alters the coupling within and between networks of bio-physical oscillators, tuning their intrinsic resonant properties to structures in the rearing environment. When combined with observations and assumptions about developmental trajectories and neural substrates of musical rhythm, we claim that *resonance* and *attunement* are powerful constructs that account for and predict key aspects of rhythm development in humans, explaining, for instance, why developmental and learning processes might favor certain rhythmic structures.

### A Developmental Model of Rhythm Perception and Action

**FIGURE 1:**
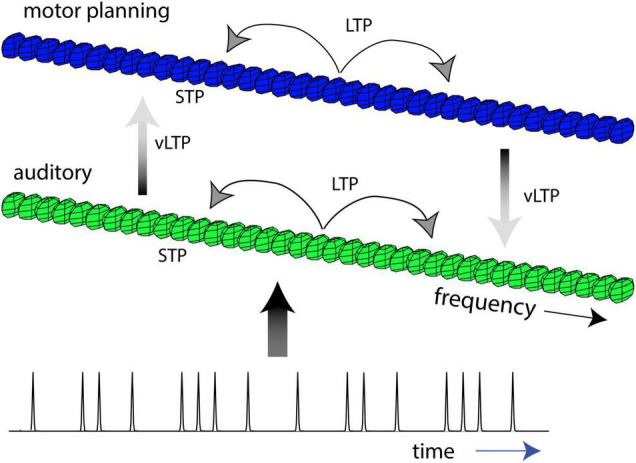
A developmental framework for rhythm perception and action. We theorize that musical rhythm development occurs over three timescales: (1) On the timescale of rhythmic patterns, the auditory network entrains and quickly adapts to complex patterns (short-term plasticity–STP). (2) On a timescale of months, infants attune to the complex rhythmic patterns they encounter in their environment (long-term plasticity–LTP). (3) On a timescale of years, children develop an adult-like ability to synchronize to acoustic rhythms (very long-term plasticity–vLTP). These processes are described *via* Hebbian plasticity dynamics with different time constants. Mature rhythm perception and action involves coupling of oscillations in the auditory network, coupling of oscillations in the motor-planning networks, and interactions between the auditory and motor networks.

Building on prior modeling work of adult rhythm perception and action (Velasco and Large, 2011; Large et al., 2015), we describe an extension to the NRT framework that enables modeling of several aspects of rhythm development (Figure 1). Currently, our developmental framework assumes two oscillatory networks (Velasco and Large, 2011; Large et al., 2015; Tichko et al., 2021), though more networks could be added, which we refer to as *auditory* and *motor-planning* networks, respectively. The oscillatory networks contain neural oscillators with different natural frequencies, enabling the auditory and motor-planning networks to represent multiple, hierarchically nested timescales in their intrinsic dynamics. Adult-like rhythm perception and action is hypothesized to involve the coupling of oscillations within the auditory network, coupling of oscillations within the motor-planning network, and coupling of oscillations between the auditory and motor-planning networks. This two-network model is sufficient to simulate the emergence of pulse perception in complex rhythms (Velasco and Large, 2011) and predicts both behavioral (Large et al., 2015) and neural entrainment (Tal et al., 2017; Large et al., 2018) to complex rhythms in adults. In addition to auditory and motor-planning networks, other layers, in theory, could also be added to the model to represent additional sensory systems, such as the visual system.

We model the development of rhythm under NRT by combining the resonant properties of the auditory and motor-planning networks with Hebbian plasticity, using a Hebbian learning rule that enables oscillatory networks to self-organize by dynamically tuning their coupling connections (Kim and Large, 2021). Hoppensteadt and Izhikevich (1996b) originally derived a Hebbian learning rule for single-frequency oscillatory networks from an underlying physiological model. Following their derivation, the Hebbian plasticity rule has been extended to support learning in oscillatory networks featuring multiple frequencies (Kim and Large, 2021). This Hebbian plasticity rule constitutes a dynamical memory that allows multi-frequency networks to learn and remember complex rhythmic patterns *via* changes in the amplitudes and phases of coupling coefficients. Similar to classical neural networks models (e.g., connectionist models), the amplitude component is akin to a connection weight and can be interpreted as the strength of the synaptic connection. Unlike classical neural networks, the coupling coefficient also contains a phase component, which enables the networks to learn and retain phase information (Kim and Large, 2021). Developmental change and perceptual learning emerge, thus, as changes in the amplitudes and phases of these coupling coefficients over ontogeny (Hoppensteadt and Izhikevich, 1996b; Tichko and Large, 2019; Kim and Large, 2021; Tichko et al., 2021). Additionally, the Hebbian plasticity rule contains a timescale parameter that determines how fast or how slowly the oscillator connections adapt to rhythmic inputs. Because the model is a scale-free canonical model, the timescale parameter can be chosen based on empirical considerations. For instance, a small value might be used to model short-term plasticity (e.g., Tichko et al., 2021) while a larger value might be used to model long-term plasticity (e.g., Tichko and Large, 2019).

The architecture of the developmental model is based on assumptions about sensorimotor development and the timescales of developmental plasticity. Beginning in the infancy period, we theorize that the auditory and motor-planning networks are initially linked with a form of developmentally nascent auditory-motor coupling. This coupling is hypothesized to be weak, diffuse, and bi-directional, resulting in a model architecture of fully connected auditory and motor-planning networks with non-specific coupling and small coupling amplitudes. Over the course of development, we theorize that coupling within and between the auditory and motor-planning networks are dynamically tuned to rhythmic inputs in the rearing environment, either growing stronger (i.e., larger amplitude components of the coupling coefficients) or becoming pruned away (i.e., smaller amplitude components of the coupling coefficients) as a consequence of Hebbian plasticity. Motivated by the extant literature on rhythm development, we hypothesize that such developmental resonance and attunement to rhythmic structures unfolds over three, principal timescales (Figure 1 and Table 1):

**TABLE 1:** How Neural Resonance Theory (NRT) accounts for key features of rhythm development across multiple ontogenetic timescales.

Timescale of plasticity	Timescale units	Developmental phenomena	Explained by Neural Resonance Theory
Short-Term Plasticity (STP)	Seconds	Neural entrainment to music; music-movement interactions during infancy	Non-linear resonance of bio-physical oscillators; transient coupling between bio-physical oscillators over seconds to minutes
Long-Term Plasticity (LTP)	Months	Perceptual narrowing to musical structure; learning of rhythmic motor patterns	Stable coupling within the auditory and motor-planning networks over months to years
Very Long-Term Plasticity (vLTP)	Years	Emergence of flexible sensorimotor synchronization to culture-specific musical rhythms across musical tempi over the lifespan	Stable coupling between auditory and motor-planning networks over years to decades

1) Short-term (STP): On the timescale of seconds, oscillations in the auditory network quickly entrain to the onsets of exogenous rhythms, resonating to complex rhythmic patterns in the environment. Further, short-term attunement occurs *via* transient coupling between oscillations that emerges and then decays over short-time scales, such as seconds and minutes, allowing the network to quickly tune their connections to repeating patterns.
2) Long-term (LTP): On a timescale of months, infants’ perceptual systems attune to the complex rhythmic patterns that constitute the organism-environment system. Attunement happens as frequency and phase relationships are learned in the auditory network *via* Hebbian plasticity. In parallel, complex motor rhythms are learned in the motor-planning network. Attunement predicts that neural and organismal embodiments of commonly encountered patterns become more stable, but less frequently encountered rhythms may be perceptually distorted, explaining infants’ perceptual narrowing to musical rhythm (Tichko and Large, 2019) by 12 months of age (Hannon and Trehub, 2005a,b).
3) Very long-term (vLTP): Over timescales of years, auditory-motor coupling is tuned and strengthened, producing a frequency-specific topography of connectivity between auditory and motor-planning networks. We propose that this topography will reflect structures common to the infants’ respective musical cultures, such as connections between oscillators in the auditory and motor-planning networks that reflect 1:1 and 2:1 ratios for Western infants. Stable coupling between auditory and motor-planning networks enables children and adults to synchronize behavior across a broad range of rhythmic patterns, yet ones that are specific to their respective cultures.

### Explaining Three Features of Rhythm Development With Neural Resonance Theory

In what follows, we discuss how our hypothesized developmental model accounts for three prominent features of rhythm development reviewed above—(1) developmental plasticity, (2) biases and constraints, and (3) longitudinal change. We, then, conclude with several, novel predictions regarding the ontogeny of rhythm derived from the model.

#### Feature 1: Developmental Plasticity to Musical Rhythm Under NRT

A key feature of rhythm development is the remarkable degree of plasticity in which infants’ and children’s perceptual systems become attuned to rhythmic patterns. This process, called “perceptual narrowing” or “perceptual fine-tuning” (Flom, 2014), suggests that infants’ perceptual symptoms gradually become fine-tuned to prominent rhythmic structures that constitute the rearing environment, as a result of experience-dependent plasticity (Hannon and Trehub, 2005b; Soley and Hannon, 2010). In a recent model, we attempted to account for infants’ perceptual fine-tuning to native musical rhythmic structures using the NRT framework (Tichko and Large, 2019). First, we employed an auditory network without internal connections to represent a developmentally younger infant (i.e., a less musically enculturated infant). In this model, we found that the oscillatory activity in the network veridically embodied a Western and Non-Western musical rhythm, reproducing the musical events and accents of each rhythm, in a manner akin to a Fourier representation of the rhythm. Next, we trained an auditory network, one equipped with Hebbian plasticity, on the Western or non-Western musical rhythm to model infants’ learning of musical rhythms. With the addition of Hebbian plasticity, we found that the amplitudes and phases of the coupling coefficients between oscillators in the auditory network became tuned, so that, together, they generated a stable, complex rhythm that reflected the structure of their respective training rhythm. Thus, the networks no longer operated veridically on the basis of individual oscillations, like a Fourier analysis; instead, the networks operated in terms of stable, learned rhythmic structures. Moreover, the networks trained on a musical rhythm were found to be biased toward musical rhythms that have the same structure as the rhythm they had learned (Tichko and Large, 2019). (To return to the comparison to frequency analysis, one could say that the neural system learns a new basis function that embodies the complex structure of the training rhythms, as a result of learned coupling between network oscillators.) Together, our simulations suggested that young infants initially possess a perceptual system that functions in a near veridical fashion (i.e., a Fourier-like embodiment of rhythm), during nascent stages of development, before becoming attuned to rhythmic structures through the self-organization of oscillator coupling.

#### Feature 2: Rhythmic Biases and Constraints Under NRT

A secondary finding on rhythm development, and one that is often contrasted against the developmental plasticity of musical rhythm, is that certain rhythmic structures, those characterized by simpler integer-ratio relationships, appear to be more easily learned, tracked, and reproduced relative to more complex rhythms (Drake, 1993; Hannon et al., 2011; Ullal-Gupta et al., 2014; Jacoby and McDermott, 2017). Such findings have led to proposals that there are intrinsic biases or constraints on rhythm perception and action. How might NRT account for these structural biases and constraints on the development of rhythm perception and action? NRT posits that biases and constraints on rhythm perception and action emerge lawfully from the universal, physical properties of non-linear oscillators (Kim and Large, 2015, 2019). For instance, from the physics of coupled oscillatory systems, biases and constraints could reflect the greater stability for coupled oscillators or oscillators coupled to external rhythms that reflect smaller integer-ratio frequency relationships. As the complexity of integer-ratio frequency relationships between oscillators or exogenous rhythms increases, the stability of the coupling decreases (Kim and Large, 2015, 2019), which may explain why, first, simple integer-ratio relationships are more prevalent in the world’s music (i.e., greater stability leads to greater prevalence), and, second, why rhythms characterized by simple integer-ratio are learned more readily during development (i.e., greater stability increases learnability). Further, the simplicity and complexity of integer-ratio relationships are related to the dynamics of learning. Our analysis found that multi-frequency Hebbian learning leads to stronger connections for simple frequency ratios and that the minimum learning rate required to achieve learning is smaller for simple ratios (Kim and Large, 2021). This suggests that, after accounting for all other developmental variables, simple rhythmic patterns based on small integer ratios are learned more readily than more complex patterns. While this general principle of non-linear resonance places constraints on what can be learned, it does not solely determine what is learned in individual experience. A moderately complex rhythmic pattern can form a stronger memory than simpler patterns, if, for instance, it is experienced more often. This is because the strength of learning depends not only on the simplicity of frequency ratio but also on the length of exposure (Kim and Large, 2021).

Our analyses which predict that simpler rhythmic structures are learned with greater rapidity have been borne out in recent modeling efforts of rhythm development. In a recent model of auditory-vestibular interactions underlying infant rhythm perception, Tichko et al. (2021) found that a NRT model learned simpler rhythmic structures (e.g., 2:1) more readily, relative to more complex rhythmic structures (e.g., 3:1). In particular, we found that NRT models learned stronger oscillator connections in the auditory network at a faster rate for rhythmic structures that followed a duple metrical relationship to the beat level (2:1), relative to a ternary metrical relationship to the beat level (3:1). This behavior of the model is consistent with recent theoretical analyses of multi-frequency Hebbian learning (Kim and Large, 2021) and with previous findings regarding infants’, children’s, and adults’ preferences and superior performance for simpler integer ratios in music (Drake, 1993; Hannon et al., 2011; Ullal-Gupta et al., 2014; Jacoby and McDermott, 2017).

#### Feature 3: Longitudinal Rhythm Development Under NRT

A final prominent characteristic of musical rhythm development is the lifespan change in both behavioral synchronization to musical rhythm and spontaneous motor tempo (SMT). As reviewed previously, the ability to synchronize behaviorally to musical rhythm gradually increases over development, while STM decreases across the lifespan (Drake et al., 2000; McAuley et al., 2006). NRT explains these two processes, respectively, as a result of the gradual coupling of auditory and motor-planning systems over ontogeny and through frequency-dependent Hebbian learning dynamics. Firstly, NRT explains the developmental changes in behavioral synchronization of musical rhythm as changes in the cross-network coupling between auditory and motor-planning networks in our developmental framework (Figure 1). In early infancy, we theorize that auditory and motor-planning networks are initially diffusely coupled, accounting for nascent auditory-motor-vestibular interactions during rhythm perception (Phillips-Silver and Trainor, 2005). By young adulthood, however, auditory and motor-planning networks are now coupled with cross-network connections that enable behavioral synchronization to culture-specific musical structures (Drake et al., 2000; McAuley et al., 2006; Kirschner and Ilari, 2014). Cross-network connections between auditory and motor-planning networks continue to stabilize into adulthood, enabling synchronization over a wide-range of rhythmic stimuli, though biasing rhythmic perception-action to rhythmic structures found in one’s native musical systems (Hannon et al., 2012).

Secondly, NRT explains the gradual decrease in tempo preferences and SMT over the lifespan (Drake et al., 2000; McAuley et al., 2006) as result of *frequency-dependent* Hebbian plasticity that arises naturally from networks featuring *multiple* frequencies. The embodiment of multiple timescales in the networks posited by NRT consequently produces faster learning dynamics for neural oscillators with a faster natural frequency, relative to oscillators with slower, intrinsic timescales. This results in faster neural oscillators coming online and forming stronger cross-network connections between other faster oscillators during earlier periods of development. Such frequency-dependent learning may explain why tempo preferences and SMT gradually slow over the lifespan, as, by late adulthood, oscillator connections between the slowest oscillators finally emerge, engendering a preference for slower tempi rhythms.

## Predictions and Conclusion

In the current paper, we have outlined a general, theoretical framework for explaining and modeling the development of musical rhythm abilities in humans. Our developmental model, grounded in Neural Resonance Theory (NRT), proposes that ontogenetic changes in rhythm perception and action occur *via* the *resonance* and the *attunement* of coupled auditory-motor systems and rhythmic inputs over development. Already, the principles of resonance and attunement, as formalized in the NRT framework, have begun to explain key facets of rhythm development, such as adults’ pulse perception to complex rhythms (Velasco and Large, 2011; Large et al., 2015), perceptual fine-tuning to culture-specific musical structures (Tichko and Large, 2019), and auditory-motor-vestibular interactions underlying rhythm perception (Tichko et al., 2021).

It is worth noting that our approach to rhythm development differs from classical theories of rhythm cognition and rhythm development that adopt an information-processing perspective. For instance, one popular approach, called statistical learning, posits that structural regularity arises from the organism’s learning of statistics that are present in environmental inputs (Aslin, 2017). While there is evidence for statistical learning of linguistic and music structure (Saffran et al., 1996; Thiessen and Saffran, 2003; Creel et al., 2004; Hannon and Johnson, 2005), statistical learning theories do not explain structural constraints in cognition and behavior, except in a circular way–lawful structures are found in cognition because they arise in behavior. In contrast to statistical learning theories, NRT does not assume that structural regularity emerges from the statistical analysis of environmental input. Rather, NRT predicts that structures emerge in a lawful manner–from a physics at the ecological scale–and provides a mechanistic account of how such structures arise and are tuned during learning and development. Indeed, unlike statistical learning theory, NRT explains why some structures can be learned, or are learned more easily relative to other kinds of structures.

To conclude, we outline several predictions generated from NRT regarding the development of musical rhythm that should be tested empirically. Perhaps the most significant aspect of the NRT approach is the ability to generate predictions about rhythm cognition and behavior from a mathematical analysis of a non-linear dynamical model of the system under study (Kim and Large, 2015, 2019, 2021). Further, because the NRT models are derived from an underlying physiological model, it is reasonable to treat the predictions generated from the model as predictions about oscillations in electroencephalography (EEG) and magnetoencephalography (MEG) measurements.

One series of predictions derived from NRT pertains to the development of neural responses to musical rhythm over the lifespan. In a recent model of adult beat perception, we tested whether neural resonance is sufficient to explain the computation of beat (and meter) from very complex rhythmic patterns [cf., Patel and Iversen (2014) and (Rimmele et al., 2018)]—specifically, rhythms that have no spectral energy at the frequency humans perceive as the beat. These rhythms are called “missing pulse” rhythms. The model hypothesized that adult beat perception, including adults’ perception of beat in missing-pulse rhythms, emerges from interactions between two oscillatory networks–an auditory network and a motor-planning network. Further, these auditory and motor-planning networks were assumed to be coupled in a manner to reflect the rhythmic structure of Western music, representing an adult-like nervous system of an enculturated listener to Western music. From simulations of this model, we observed that the auditory network tended to embody complex rhythmic stimuli more-or-less veridically, while the pulse emerged in the motor-planning network. Importantly, our model predicted that neural oscillations would emerge at the “missing” pulse frequency in response to missing-pulse rhythms (Velasco and Large, 2011; Large et al., 2015).

The behavior of the model was, then, used to predict behavioral and neural responses in adult humans to these same very complex rhythms. Firstly, we found that adult listeners perceive and synchronize at missing pulse frequencies and that the phase of participant responses was multi-stable, as predicted by our model (Large et al., 2015). Secondly, several EEG and MEG studies later reported missing pulse responses in auditory brain areas (Nozaradan et al., 2012; Cirelli et al., 2016; Tal et al., 2017), consistent with the behavior of our model. In our most recent EEG study (Wasserman, 2018), we observed (1) strong pulse-frequency brain responses to isochronous and missing pulse rhythms, but not to a random control; (2) strong coherence between brain responses and model-predicted auditory and motor neural responses; and (3) different pulse-frequency topographies for missing pulse rhythms (versus isochronous and random). These results support the theory that beat perception occurs as the result of an emergent population oscillation that entrains at the pulse frequency, possibly in motor networks of the brain (Wasserman, 2018).

In addition to our adult model, our hypothesized developmental model could also be used to predict neural responses to complex “missing pulse” rhythms across the lifespan. As our adult model of pulse perception (Large et al., 2015) already predicts that stable connections between auditory and motor-planning systems are needed to perceive the beat, especially for rhythmic stimuli in which the beat is physically absent from the acoustics of the rhythm (i.e., syncopated and “missing-pulse” rhythms), we, thus, predict during early stages of development, where auditory-motor coupling is weak, neural responses at the missing pulse frequency may not be easily observed. However, as coupling between auditory and motor-planning systems strengths over development, neural responses to missing-pulse rhythms should become more evident. Thus, we predict that the strength of coupling between auditory and motor-planning networks, represented as the amplitude component of coupling coefficients in our model, will track with the emergence of neural responses to missing-pulse rhythms. Further, we predict that neural responses to missing-pulse rhythms, which may index auditory-motor coupling, would track with children’s development of *behavioral* synchronization to rhythm, as behavioral synchronization is also posited to depend on auditory-motor coupling. This developmental process could be modeled as our hypothesized very-long-term Hebbian plasticity (vLTP) process that gradually couples auditory and motor-planning networks over the lifespan, with different strengths of auditory-motor coupling reflecting different stages of sensorimotor development. Finally, we predict that aberrant patterns of auditory-motor coupling might manifest as particular musical disorders: beat-deafness (Phillips-Silver et al., 2011), for instance, is a disorder in which listeners have difficulty perceiving and coordinating action to music. From the NRT perspective, beat deafness may arise from weak or aberrant coupling between auditory and motor-planning neural systems (Sowiński and Dalla Bella, 2013).

A related prediction concerns the veridical embodiment of musical rhythm in EEG and MEG activity. Early in development, the neural response to auditory rhythm is hypothesized to be nearly veridical (Tichko and Large, 2019), explaining the ability of 6-month-old infants to detect temporal disruptions to native and non-native rhythms and meters (Hannon and Trehub, 2005b). While data regarding infants’ neural responses to musical rhythm is limited (Winkler et al., 2009; Cirelli et al., 2016), near-veridical representations of musical rhythm have recently been observed in infants’ electroencephalogram (EEG) (Cirelli et al., 2016). Consistent with our modeling, near veridical neural responses have been observed when adults listen, but do not mentally group, an acoustic rhythm (Nozaradan et al., 2011), when adults do not understand the language (Zou et al., 2019), and, in infants, who have not yet had significant auditory exposure to rhythms (Cirelli et al., 2016). Unfortunately, at the EEG-MEG level, a veridical oscillatory response is indistinguishable from the frequency analysis of a train of evoked potentials. Therefore, researchers have tended to focus on metrical frequencies that can be observed in adults in auditory cortex under the right experimental paradigms (Tal et al., 2017). Recent work, however, suggests that neural responses to musical rhythm are more consistent with an oscillator model than an evoked-potential model (Doelling et al., 2019), lending further support that neural responses to music rhythm, as posited by NRT, may involve oscillatory dynamics.

A second line of predictions from our hypothesized developmental model concerns the learnability of different rhythmic structures. Our analysis of Hebbian plasticity in multi-frequency oscillatory networks indicated that time-varying inputs with simple frequency ratios are easier to learn, relative to time-varying inputs characterized by more complex frequency ratios (Kim and Large, 2021). Thus, NRT predicts that simpler rhythmic structures, particularly those characterized by simpler integer ratios, can be learned more readily and with less exposure than more complex rhythmic structures over human development. While NRT predicts that simpler rhythmic structures can be learned with greater rapidity, NRT also predicts that complex rhythmic structures, such as those found in Non-Western music, can also be learned by networks of neural oscillators (Tichko and Large, 2019), but may require more training and experience (Kim and Large, 2021).

Finally, our developmental model could be used to predict how short-term (e.g., maternal bouncing), long-term (e.g., infant music classes), and very long-term (e.g., exposure to one’s native music, musical training) learning dynamics influence rhythmic abilities. Training our proposed developmental model on different classes of musical rhythms for differing training periods (i.e., different timescale parameters) could be done to generate predictions regarding neural and behavioral measures of rhythm abilities across these multiple timescales.

## Author Contributions

PT, JK, and EL developed the theoretical framework and wrote the initial review of Neural Resonance Theory and dynamical systems theory. PT wrote the initial review of rhythm development. EL created the figure. All authors read and edited the manuscript and approved the final manuscript.

## Conflict of Interest

The authors declare that the research was conducted in the absence of any commercial or financial relationships that could be construed as a potential conflict of interest.

## Publisher’s Note

All claims expressed in this article are solely those of the authors and do not necessarily represent those of their affiliated organizations, or those of the publisher, the editors and the reviewers. Any product that may be evaluated in this article, or claim that may be made by its manufacturer, is not guaranteed or endorsed by the publisher.

## Footnotes

1 Our use of “resonance” is derived from dynamical systems theory to mean non-linear resonance—a general term that includes entrainment, synchronization, phase-locking, and mode-locking, among other phenomena. For characterization of some of the many different dynamical systems that exhibit non-linear resonance, see Kim and Large (2015, 2019, 2021).

## References

[B1] Aslin R. N. (2017). Statistical learning: a powerful mechanism that operates by mere exposure. *Wiley Interdiscip. Rev. Cogn. Sci.* 8:e1373. 10.1002/wcs.1373 27906526 PMC5182173

[B2] Bengtsson S. L. Ullén F. Ehrsson H. H. Hashimoto T. Kito T. Naito E. (2009). Listening to rhythms activates motor and premotor cortices. *Cortex* 45 62–71. 10.1016/j.cortex.2008.07.002 19041965

[B3] Bergeson T. R. Trehub S. E. (2006). Infants perception of rhythmic patterns. *Music Percept.* 23 345–360. 10.1525/mp.2006.23.4.345

[B4] Bobin-Bègue A. Provasi J. (2005). Tempo discrimination in 3- and 4-year-old children: performances and threshold. *Curr. Psychol. Lett.* 60 611–624. 10.4000/cpl.440

[B5] Busnel M. C. (1979). Intravaginal measurements of the level and acoustic distortion of maternal noises. *Electrodiagn. Ther.* 16:142. 535572

[B6] Cannon J. J. Patel A. D. (2021). How beat perception co-opts motor neurophysiology. *Trends Cogn. Sci.* 25 137–150. 10.1016/j.tics.2020.11.002 33353800 PMC9440376

[B7] Chang H. W. Trehub S. E. (1977). Infants’ perception of temporal grouping in auditory patterns. *Child Dev.* 48 1666–1670. 608377

[B8] Chemero A. (2013). Radical embodied cognitive science. *Rev. Gen. Psychol.* 17 145–150.

[B9] Chen J. L. Penhune V. B. Zatorre R. J. (2008). Listening to musical rhythms recruits motor regions of the brain. *Cereb. Cortex* 18 2844–2854. 10.1093/cercor/bhn042 18388350

[B10] Cirelli L. K. Spinelli C. Nozaradan S. Trainor L. J. (2016). Measuring neural entrainment to beat and meter in infants: effects of music background. *Front. Neurosci.* 10:229. 10.3389/fnins.2016.00229 27252619 PMC4877507

[B11] Collier G. L. Wright C. E. (1995). Temporal rescaling of simple and complex ratios in rhythmic tapping. *J. Exp. Psychol. Hum. Percept. Perform.* 21 602–627. 10.1037//0096-1523.21.3.602 7790836

[B12] Cooper G. W. Meyer L. B. (1963). *The Rhythmic Structure of Music.* Chicago, IL: University of Chicago Press.

[B13] Creel S. C. Newport E. L. Aslin R. N. (2004). Distant melodies: statistical learning of nonadjacent dependencies in tone sequences. *J. Exp. Psychol. Learn. Mem. Cogn.* 30 1119–1130. 10.1037/0278-7393.30.5.1119 15355140

[B14] DeCasper A. J. Fifer W. P. (1980). Of human bonding: newborns prefer their mothers’ voices. *Science* 208 1174–1176. 10.1126/science.7375928 7375928

[B15] DeCasper A. J. Sigafoos A. D. (1983). The intrauterine heartbeat: a potent reinforcer for newborns. *Infant Behav. Dev.* 6 19–25. 10.1016/S0163-6383(83)80004-6

[B16] DeCasper A. J. Spence M. J. (1986). Prenatal maternal speech influences newborns’ perception of speech sounds. *Infant Behav. Dev.* 9 133–150. 7981479

[B17] Demany L. McKenzie B. Vurpillot E. (1977). Rhythm perception in early infancy. *Nature* 266 718–719. 10.1038/266718a0 876350

[B18] Doelling K. B. Assaneo M. F. Bevilacqua D. Pesaran B. Poeppel D. (2019). An oscillator model better predicts cortical entrainment to music. *Proc. Natl. Acad. Sci. U.S.A.* 116 10113–10121. 10.1073/pnas.1816414116 31019082 PMC6525506

[B19] Doelling K. B. Poeppel D. (2015). Cortical entrainment to music and its modulation by expertise. *Proc. Natl. Acad. Sci. U.S.A.* 112 E6233–E6242. 10.1073/pnas.1508431112 26504238 PMC4653203

[B20] Doheny L. Hurwitz S. Insoft R. Ringer S. Lahav A. (2012). Exposure to biological maternal sounds improves cardiorespiratory regulation in extremely preterm infants. *J. Matern. Fetal Neonatal Med.* 25 1591–1594. 10.3109/14767058.2011.648237 22185623

[B21] Dotov D. G. (2014). Putting reins on the brain. how the body and environment use it. *Front. Hum. Neurosci.* 8:795. 10.3389/fnhum.2014.00795 25346675 PMC4191179

[B22] Drake C. (1993). Reproduction of musical rhythms by children, adult musicians, and adult nonmusicians. *Percept. Psychophys.* 53 25–33. 10.3758/bf03211712 8433903

[B23] Drake C. (1997). Motor and perceptually preferred synchronisation by children and adults: binary and ternary ratios. *Pol. Q. Dev. Psychol*. 3 41–59.

[B24] Drake C. Jones M. R. Baruch C. (2000). The development of rhythmic attending in auditory sequences: attunement, referent period, focal attending. *Cognition* 77 251–288. 10.1016/s0010-0277(00)00106-2 11018511

[B25] Eerola T. Luck G. Toiviainen P. (2006). “An investigation of pre-schoolers’ corporeal synchronization with music,” in *Proceedings of the 2006 9th International Conference on Music Perception and Cognition*, Bologna, 472–476.

[B26] Einarson K. M. Trainor L. J. (2016). Hearing the Beat. *Music Percept.* 34 56–70. 10.1525/mp.2016.34.1.56

[B27] Fifer W. P. Moon C. M. (1994). The role of mother’s voice in the organization of brain function in the newborn. *Acta Paediatr. Suppl.* 397 86–93. 10.1111/j.1651-2227.1994.tb13270.x 7981479

[B28] Flom R. (2014). Perceptual narrowing: retrospect and prospect. *Dev. Psychobiol.* 56 1442–1453. 10.1002/dev.21238 25042698

[B29] Fraisse P. (1982). “Rhythm and Tempo,” in *The Psychology of Music*, ed. Deutsch D. (Orlando, FL: Academic).

[B30] Frank T. D. Daffertshofer A. Peper C. E. Beek P. J. Haken H. (2000). Towards a comprehensive theory of brain activity: coupled oscillator systems under external forces. *Physica D* 144 62–86.

[B31] Fujii S. Watanabe H. Oohashi H. Hirashima M. Nozaki D. Taga G. (2014). Precursors of dancing and singing to music in three- to four-months-old infants. *PLoS One* 9:e97680. 10.1371/journal.pone.0097680 24837135 PMC4023986

[B32] Fujioka T. Ross B. Trainor L. J. (2015). Beta-Band oscillations represent auditory beat and its metrical hierarchy in perception and imagery. *J. Neurosci.* 35 15187–15198. 10.1523/JNEUROSCI.2397-15.2015 26558788 PMC6605356

[B33] Fujioka T. Trainor L. J. Large E. W. Ross B. (2009). Beta and gamma rhythms in human auditory cortex during musical beat processing. *Ann. N. Y. Acad. Sci.* 1169 89–92. 10.1111/j.1749-6632.2009.04779.x 19673759

[B34] Fujioka T. Trainor L. J. Large E. W. Ross B. (2012). Internalized timing of isochronous sounds is represented in neuromagnetic β oscillations. *J. Neurosci.* 32 1791–1802. 10.1523/JNEUROSCI.4107-11.2012 22302818 PMC6703342

[B35] Gerry D. W. Faux A. L. Trainor L. J. (2010). Effects of kindermusik training on infants’ rhythmic enculturation. *Dev. Sci.* 13 545–551. 10.1111/j.1467-7687.2009.00912.x 20443974

[B36] Gibson J. J. (1966). *The Senses Considered as Perceptual Systems.* Boston, MA: Houghton Mifflin. Available online at: https://psycnet.apa.org/fulltext/1966-35026-000.pdf (accessed January 6, 2021).

[B37] Giovanelli G. Sansavini A. Farneti A. (1999). “Perception of sound, rhythm and speech from pre-natal to post-natal life,” in *Current Issues in Developmental Psychology: Biopsychological Perspectives*, eds Kalverboer A. F. Genta M. L. Hopkins J. B. (Dordrecht: Springer), 137–159. 10.1007/978-94-011-4507-7_6

[B38] Gordon C. L. Cobb P. R. Balasubramaniam R. (2018). Recruitment of the motor system during music listening: an ALE meta-analysis of fMRI data. *PLoS One* 13:e0207213. 10.1371/journal.pone.0207213 30452442 PMC6242316

[B39] Grahn J. A. Brett M. (2007). Rhythm and beat perception in motor areas of the brain. *J. Cogn. Neurosci.* 19 893–906. 10.1162/jocn.2007.19.5.893 17488212

[B40] Grahn J. A. Rowe J. B. (2009). Feeling the beat: premotor and striatal interactions in musicians and nonmusicians during beat perception. *J. Neurosci.* 29 7540–7548. 10.1523/JNEUROSCI.2018-08.2009 19515922 PMC2702750

[B41] Granier-Deferre C. Bassereau S. Ribeiro A. Jacquet A.-Y. Decasper A. J. (2011a). A melodic contour repeatedly experienced by human near-term fetuses elicits a profound cardiac reaction one month after birth. *PLoS One* 6:e17304. 10.1371/journal.pone.0017304 21383836 PMC3044162

[B42] Granier-Deferre C. Ribeiro A. Jacquet A.-Y. Bassereau S. (2011b). Near-term fetuses process temporal features of speech. *Dev. Sci.* 14 336–352. 10.1111/j.1467-7687.2010.00978.x 22213904

[B43] Haken H. Kelso J. A. Bunz H. (1985). A theoretical model of phase transitions in human hand movements. *Biol. Cybern.* 51 347–356. 10.1007/BF00336922 3978150

[B44] Haken H. Peper C. E. Beek P. J. Daffertshofer A. (1996). A model for phase transitions in human hand movements during multifrequency tapping. *Physica D* 90 179–196. 10.1016/0167-2789(95)00235-9

[B45] Hannon E. E. Johnson S. P. (2005). Infants use meter to categorize rhythms and melodies: implications for musical structure learning. *Cogn. Psychol.* 50 354–377. 10.1016/j.cogpsych.2004.09.003 15893524

[B46] Hannon E. E. Nave-Blodgett J. E. Nave K. M. (2018). The developmental origins of the perception and production of musical rhythm. *Child Dev. Perspect.* 12 194–198. 10.1111/cdep.12285

[B47] Hannon E. E. Soley G. Levine R. S. (2011). Constraints on infants’ musical rhythm perception: effects of interval ratio complexity and enculturation. *Dev. Sci.* 14 865–872. 10.1111/j.1467-7687.2011.01036.x 21676105

[B48] Hannon E. E. Trehub S. E. (2005a). Metrical categories in infancy and adulthood. *Psychol. Sci.* 16 48–55. 10.1111/j.0956-7976.2005.00779.x 15660851

[B49] Hannon E. E. Trehub S. E. (2005b). Tuning in to musical rhythms: infants learn more readily than adults. *Proc. Natl. Acad. Sci. U.S.A.* 102 12639–12643. 10.1073/pnas.0504254102 16105946 PMC1194930

[B50] Hannon E. E. Vanden Bosch der Nederlanden C. M. Tichko P. (2012). Effects of perceptual experience on children’s and adults’ perception of unfamiliar rhythms. *Ann. N. Y. Acad. Sci.* 1252 92–99. 10.1111/j.1749-6632.2012.06466.x 22524345

[B51] Harding E. E. Sammler D. Henry M. J. Large E. W. Kotz S. A. (2019). Cortical tracking of rhythm in music and speech. *Neuroimage* 185 96–101. 10.1016/j.neuroimage.2018.10.037 30336253

[B52] Hepper P. G. (1991). An examination of fetal learning before and after birth. *Ir. J. Psychol.* 12 95–107. 10.1080/03033910.1991.10557830

[B53] Hoppensteadt F. C. Izhikevich E. M. (1996a). Synaptic organizations and dynamical properties of weakly connected neural oscillators. I. Analysis of a canonical model. *Biol. Cybern.* 75 117–127. 10.1007/s004220050279 8855350

[B54] Hoppensteadt F. C. Izhikevich E. M. (1996b). Synaptic organizations and dynamical properties of weakly connected neural oscillators II. Learning phase information. *Biol. Cybern.* 75 129–135. 10.1007/s004220050280 8855351

[B55] Ilari B. (2015). Rhythmic engagement with music in early childhood: a replication and extension. *J. Res. Music Educ.* 62 332–343. 10.1177/0022429414555984

[B56] Jacoby N. McDermott J. H. (2017). Integer ratio priors on musical rhythm revealed cross-culturally by iterated reproduction. *Curr. Biol.* 27 359–370. 10.1016/j.cub.2016.12.031 28065607

[B57] James D. K. Spencer C. J. Stepsis B. W. (2002). Fetal learning: a prospective randomized controlled study. *Ultrasound Obstet. Gynecol.* 20 431–438. 10.1046/j.1469-0705.2002.00845.x 12423478

[B58] Kim J. C. Large E. W. (2015). Signal processing in periodically forced gradient frequency neural networks. *Front. Comput. Neurosci.* 9:152. 10.3389/fncom.2015.00152 26733858 PMC4689852

[B59] Kim J. C. Large E. W. (2019). Mode locking in periodically forced gradient frequency neural networks. *Phys. Rev. E* 99:022421. 10.1103/PhysRevE.99.022421 30934299

[B60] Kim J. C. Large E. W. (2021). Multifrequency hebbian plasticity in coupled neural oscillators. *Biol. Cybern.* 115 43–57. 10.1007/s00422-020-00854-6 33399947

[B61] Kirschner S. Ilari B. (2014). Joint drumming in Brazilian and German preschool children: cultural differences in rhythmic entrainment, but no prosocial effects. *J. Cross. Cult. Psychol.* 45 137–166. 10.1177/0022022113493139

[B62] Kirschner S. Tomasello M. (2010). Joint music making promotes prosocial behavior in 4-year-old children. *Evol. Hum. Behav.* 31 354–364. 10.1016/j.evolhumbehav.2010.04.004

[B63] Kisilevsky B. S. Hains S. M. J. Lee K. Xie X. Huang H. Ye H. H. (2003). Effects of experience on fetal voice recognition. *Psychol. Sci.* 14 220–224. 10.1111/1467-9280.02435 12741744

[B64] Kisilevsky S. Hains S. M. J. Jacquet A. Y. Granier-Deferre C. Lecanuet J. P. (2004). Maturation of fetal responses to music. *Dev. Sci.* 7 550–559. 10.1111/j.1467-7687.2004.00379.x 15603288

[B65] Kirschner S. Tomasello M. (2009). Joint drumming: social context facilitates synchronization in preschool children. *J. Exp. Child Psychol.* 102 299–314. 10.1016/j.jecp.2008.07.005 18789454

[B66] Kung S.-J. Chen J. L. Zatorre R. J. Penhune V. B. (2013). Interacting cortical and basal ganglia networks underlying finding and tapping to the musical beat. *J. Cogn. Neurosci.* 25 401–420. 10.1162/jocn_a_00325 23163420

[B67] Lahav A. Skoe E. (2014). An acoustic gap between the NICU and womb: a potential risk for compromised neuroplasticity of the auditory system in preterm infants. *Front. Neurosci.* 8:381. 10.3389/fnins.2014.00381 25538543 PMC4256984

[B68] Large E. W. (2010). “Neurodynamics of Music,” in *Music Perception*, eds Riess Jones M. Fay R. R. Popper A. N. (New York, NY: Springer), 201–231. 10.1007/978-1-4419-6114-3_7

[B69] Large E. W. Almonte F. V. Velasco M. J. (2010). A canonical model for gradient frequency neural networks. *Physica D* 239 905–911. 10.1016/j.physd.2009.11.015

[B70] Large E. W. Herrera J. A. Velasco M. J. (2015). Neural networks for beat perception in musical rhythm. *Front. Syst. Neurosci.* 9:159. 10.3389/fnsys.2015.00159 26635549 PMC4658578

[B71] Large E. W. Jones M. R. (1999). The dynamics of attending: how people track time-varying events. *Psychol. Rev.* 106 119–159.

[B72] Large E. W. Kolen J. F. (1994). Resonance and the perception of musical meter. *Conn. Sci.* 6 177–208.

[B73] Large E. W. Snyder J. S. (2009). Pulse and meter as neural resonance. *Ann. N. Y. Acad. Sci.* 1169 46–57. 10.1111/j.1749-6632.2009.04550.x 19673754

[B74] Large E. W. Wasserman C. S. Skoe E. Read H. L. (2018). Neural entrainment to missing pulse rhythms. *J. Acoust. Soc. Am.* 144 1760–1760. 28559379

[B75] Lerdahl F. Jackendoff R. (1983). *A Generative Theory of Tonal Music.* Cambridge, MA: MIT Press.

[B76] London J. (2004). *Hearing in Time: Psychological Aspects of Musical Meter.* Oxford: Oxford University Press.

[B77] Lynch M. P. Eilers R. E. (1992). A study of perceptual development for musical tuning. *Percept. Psychophys.* 52 599–608. 10.3758/bf03211696 1287565

[B78] Lynch M. P. Eilers R. E. Oller D. K. Urbano R. C. (1990). Innateness, experience, and music perception. *Psychol. Sci.* 1 272–276.

[B79] McAuley J. D. Jones M. R. Holub S. Johnston H. M. Miller N. S. (2006). The time of our lives: life span development of timing and event tracking. *J. Exp. Psychol. Gen.* 135 348–367. 10.1037/0096-3445.135.3.348 16846269

[B80] Merchant H. Honing H. (2013). Are non-human primates capable of rhythmic entrainment? Evidence for the gradual audiomotor evolution hypothesis. *Front. Neurosci.* 7:274. 10.3389/fnins.2013.00274 24478618 PMC3894452

[B81] Michaels C. F. Carello C. (1981). *Direct Perception.* Englewoodcliffs, NJ: Prentice-Hall.

[B82] Morrongiello B. A. (1984). Auditory temporal pattern perception in 6- and 12-month-old infants. *Dev. Psychol.* 20 441–448. 10.1371/journal.pone.0089275 24586651 PMC3930698

[B83] Murooka H. Koie Y. Suda N. (1976). Analysis of intrauterine sounds and their tranquillizing effects on the newborn infant. *J. Gynecol. Obstet. Biol. Reprod.* 5 367–376. 783244

[B84] Nave-Blodgett J. E. Snyder J. S. Hannon E. E. (2020). Hierarchical beat perception develops throughout childhood and adolescence and is enhanced in those with musical training. *J. Exp. Psychol. Gen.* 150 314–339. 10.1037/xge0000903 32852978

[B85] Nozaradan S. Peretz I. Missal M. Mouraux A. (2011). Tagging the neuronal entrainment to beat and meter. *J. Neurosci.* 31 10234–10240. 10.1523/jneurosci.0411-11.2011 21753000 PMC6623069

[B86] Nozaradan S. Peretz I. Mouraux A. (2012). Selective neuronal entrainment to the beat and meter embedded in a musical rhythm. *J. Neurosci.* 32 17572–17581. 10.1523/jneurosci.3203-12.2012 23223281 PMC6621650

[B87] Otte R. A. Winkler I. Braeken M. A. K. A. Stekelenburg J. J. van der Stelt O. Van den Bergh B. R. H. (2013). Detecting violations of temporal regularities in waking and sleeping two-month-old infants. *Biol. Psychol.* 92 315–322. 10.1016/j.biopsycho.2012.09.009 23046905

[B88] Parncutt R. Chuckrow R. (2019). Chuckrow’s theory of the prenatal origin of music. *Music Sci.* 23 403–425. 10.1177/1029864917738130

[B89] Patel A. D. (2010). *Music, Language, and the Brain.* Oxford: Oxford University Press.

[B90] Patel A. D. Iversen J. R. (2014). The evolutionary neuroscience of musical beat perception: the Action Simulation for Auditory Prediction (ASAP) hypothesis. *Front. Syst. Neurosci.* 8:57. 10.3389/fnsys.2014.00057 24860439 PMC4026735

[B91] Phillips-Silver J. Trainor L. J. (2005). Feeling the beat: movement influences infant rhythm perception. *Science* 308:1430. 10.1126/science.1110922 15933193

[B92] Phillips-Silver J. Trainor L. J. (2007). Hearing what the body feels: auditory encoding of rhythmic movement. *Cognition* 105 533–546. 17196580 10.1016/j.cognition.2006.11.006

[B93] Phillips-Silver J. Toiviainen P. Gosselin N. Piché O. Nozaradan S. Palmer C. (2011). Born to dance but beat deaf: a new form of congenital amusia. *Neuropsychologia* 49 961–969. 10.1016/j.neuropsychologia.2011.02.002 21316375

[B94] Povel D. J. (1981). Internal representation of simple temporal patterns. *J. Exp. Psychol. Hum. Percept. Perform.* 7 3–18. 10.1037//0096-1523.7.1.3 6452500

[B95] Proksch S. Comstock D. C. Médé B. Pabst A. Balasubramaniam R. (2020). Motor and predictive processes in auditory beat and rhythm perception. *Front. Hum. Neurosci.* 14:578546. 10.3389/fnhum.2020.578546 33061902 PMC7518112

[B96] Provasi J. Anderson D. I. Barbu-Roth M. (2014). Rhythm perception, production, and synchronization during the perinatal period. *Front. Psychol.* 5:1048. 10.3389/fpsyg.2014.01048 25278929 PMC4166894

[B97] Querleu D. Renard X. Versyp F. Paris-Delrue L. Crèpin G. (1988). Fetal hearing. *Eur. J. Obstet. Gynecol. Reprod. Biol.* 28 191–212. 3061844 10.1016/0028-2243(88)90030-5

[B98] Raja V. (2019). From metaphor to theory: the role of resonance in perceptual learning. *Adapt. Behav.* 27 405–421.

[B99] Raja V. (2020). Resonance and radical embodiment. *Synthese* 99(Suppl 1), 113–141. 10.1007/s11229-020-02610-6

[B100] Repp B. H. (2005). Sensorimotor synchronization: a review of the tapping literature. *Psychon. Bull. Rev.* 12 969–992. 10.3758/bf03206433 16615317

[B101] Repp B. H. Su Y.-H. (2013). Sensorimotor synchronization: a review of recent research (2006-2012). *Psychon. Bull. Rev.* 20 403–452. 10.3758/s13423-012-0371-2 23397235

[B102] Rimmele J. M. Morillon B. Poeppel D. Arnal L. H. (2018). Proactive sensing of periodic and aperiodic auditory patterns. *Trends Cogn. Sci.* 22 870–882. 10.1016/j.tics.2018.08.003 30266147

[B103] Rocha S. Mareschal D. (2017). Getting into the groove: the development of tempo-flexibility between 10 and 18 months of age. *Infancy* 22 540–551. 10.1111/infa.12169

[B104] Rocha S. Southgate V. H. Mareschal D. (2020). Infant spontaneous motor tempo. *Dev. Sci.* 24:e13032. 10.1111/desc.13032 32860482

[B105] Saffran J. R. Aslin R. N. Newport E. L. (1996). Statistical learning by 8-month-old infants. *Science* 274 1926–1928. 10.1126/science.274.5294.1926 8943209

[B106] Sansavini A. (1997). Neonatal perception of the rhythmical structure of speech: the role of stress patterns. *Early Dev. Parent.* 6 3–13. 10.1002/(sici)1099-0917(199703)6:1<3::aid-edp140>3.0.co;2-7

[B107] Scheurich R. Pfordresher P. Q. Palmer C. (2020). Musical training enhances temporal adaptation of auditory-motor synchronization. *Exp. Brain Res.* 238 81–92. 10.1007/s00221-019-05692-y 31792555

[B108] Scheurich R. Zamm A. Palmer C. (2018). Tapping into rate flexibility: musical training facilitates synchronization around spontaneous production rates. *Front. Psychol.* 9:458. 10.3389/fpsyg.2018.00458 29681872 PMC5897499

[B109] Schmidt R. C. Carello C. Turvey M. T. (1990). Phase transitions and critical fluctuations in the visual coordination of rhythmic movements between people. *J. Exp. Psychol. Hum. Percept. Perform.* 16 227–247. 10.1037//0096-1523.16.2.227 2142196

[B110] Scott Kelso J. A. (1995). *Dynamic Patterns: The Self-Organization of Brain and Behavior.* Cambridge, MA: MIT Press.

[B111] Slater J. Tierney A. Kraus N. (2013). At-risk elementary school children with one year of classroom music instruction are better at keeping a beat. *PLoS One* 8:e77250. 10.1371/journal.pone.0077250 24130865 PMC3795075

[B112] Smith J. (1983). “Reproduction and representation of musical rhythms: the effects of musical skill,” in *The Acquisition of Symbolic Skills*, eds Rogers D. Sloboda J. A. (Boston, MA: Springer), 273–282. 10.1007/978-1-4613-3724-9_31

[B113] Smith K. C. Cuddy L. L. (1989). Effects of metric and harmonic rhythm on the detection of pitch alterations in melodic sequences. *J. Exp. Psychol. Hum. Percept. Perform.* 15 457–471. 10.1037//0096-1523.15.3.457 2527955

[B114] Soley G. Hannon E. E. (2010). Infants prefer the musical meter of their own culture: a cross-cultural comparison. *Dev. Psychol.* 46 286–292. 10.1037/a0017555 20053025

[B115] Sowiński J. Dalla Bella S. (2013). Poor synchronization to the beat may result from deficient auditory-motor mapping. *Neuropsychologia* 51 1952–1963. 10.1016/j.neuropsychologia.2013.06.027 23838002

[B116] Tal I. Large E. W. Rabinovitch E. Wei Y. Schroeder C. E. Poeppel D. (2017). Neural entrainment to the beat: the “Missing-Pulse” Phenomenon. *J. Neurosci.* 37 6331–6341. 10.1523/JNEUROSCI.2500-16.2017 28559379 PMC5490067

[B117] Teie D. (2016). A comparative analysis of the universal elements of music and the fetal environment. *Front. Psychol.* 7:1158. 10.3389/fpsyg.2016.01158 27555828 PMC4977359

[B118] Thelen E. Smith L. B. (1996). *A Dynamic Systems Approach to the Development of Cognition and Action.* Cambridge, MA: MIT Press.

[B119] Thiessen E. D. Saffran J. R. (2003). When cues collide: use of stress and statistical cues to word boundaries by 7- to 9-month-old infants. *Dev. Psychol.* 39 706–716. 10.1037/0012-1649.39.4.706 12859124

[B120] Thompson E. Varela F. J. (2001). Radical embodiment: neural dynamics and consciousness. *Trends Cogn. Sci.* 5 418–425. 10.1016/s1364-6613(00)01750-2 11707380

[B121] Thompson E. C. White-Schwoch T. Tierney A. Kraus N. (2015). Beat synchronization across the lifespan: intersection of development and musical experience. *PLoS One* 10:e0128839. 10.1371/journal.pone.0128839 26107927 PMC4481101

[B122] Tichko P. Kim J. C. Large E. W. (2021). Bouncing the network: a dynamical systems model of auditory-vestibular interactions underlying infants’ perception of musical rhythm. *Dev. Sci.* 24:e13103. 10.1111/desc.13103 33570778

[B123] Tichko P. Large E. W. (2019). Modeling infants’ perceptual narrowing to musical rhythms: neural oscillation and Hebbian plasticity. *Ann. N. Y. Acad. Sci.* 1453 125–139. 10.1111/nyas.14050 31021447

[B124] Tichko P. Skoe E. (2018). Musical experience, sensorineural auditory processing, and reading subskills in adults. *Brain Sci.* 8:77. 10.3390/brainsci8050077 29702572 PMC5977068

[B125] Todd N. P. M. Lee C. S. (2015). The sensory-motor theory of rhythm and beat induction 20 years on: a new synthesis and future perspectives. *Front. Hum. Neurosci.* 9:444. 10.3389/fnhum.2015.00444 26379522 PMC4549635

[B126] Treffner P. J. Turvey M. T. (1993). Resonance constraints on rhythmic movement. *J. Exp. Psychol. Hum. Percept. Perform.* 19 1221–1237. 10.1037/0096-1523.19.6.1221

[B127] Trehub S. E. Hannon E. E. (2009). Conventional rhythms enhance infants’ and adults’ perception of musical patterns. *Cortex* 45 110–118. 10.1016/j.cortex.2008.05.012 19058799

[B128] Turvey M. T. (2007). Action and perception at the level of synergies. *Hum. Mov. Sci.* 26 657–697. 10.1016/j.humov.2007.04.002 17604860

[B129] Ullal-Gupta S. Hannon E. E. Snyder J. S. (2014). Tapping to a slow tempo in the presence of simple and complex meters reveals experience-specific biases for processing music. *PLoS One* 9:e102962. 10.1371/journal.pone.0102962 25075514 PMC4116158

[B130] Ullal-Gupta S. Vanden Bosch der Nederlanden C. M. Tichko P. Lahav A. Hannon E. E. (2013). Linking prenatal experience to the emerging musical mind. *Front. Syst. Neurosci.* 7:48. 10.3389/fnsys.2013.00048 24027502 PMC3759965

[B131] Upitis R. (1987). Children’s understanding of rhythm: the relationship between development and music training. *Psychomusicology* 7 41–60. 10.1037/h0094187

[B132] Velasco M. J. Large E. W. (2011). Pulse detection in syncopated rhythms using neural oscillators. *Pulse* 1 3–4.

[B133] Volman M. J. Geuze R. H. (2000). Temporal stability of rhythmic tapping “on” and “off the beat”: a developmental study. *Psychol. Res.* 63 62–69. 10.1007/pl00008168 10743387

[B134] Warren W. H. (2006). The dynamics of perception and action. *Psychol. Rev.* 113 358–389. 16637765 10.1037/0033-295X.113.2.358

[B135] Wasserman C. S. (2018). *Neural Resonance Theory: Investigating Beat-Perception Using Missing Pulse Rhythms.* Master’ thesis. Storrs, CT: University of Connecticut. Available online at: https://opencommons.uconn.edu/gs_theses/1272 (accessed January 6, 2021).

[B136] Webb A. R. Heller H. T. Benson C. B. Lahav A. (2015). Mother’s voice and heartbeat sounds elicit auditory plasticity in the human brain before full gestation. *Proc. Natl. Acad. Sci. U.S.A.* 112 3152–3157. 10.1073/pnas.1414924112 25713382 PMC4364233

[B137] Wilson H. R. Cowan J. D. (1972). Excitatory and inhibitory interactions in localized populations of model neurons. *Biophys. J.* 12 1–24. 10.1016/S0006-3495(72)86068-5 4332108 PMC1484078

[B138] Wilson M. Wilson T. P. (2005). An oscillator model of the timing of turn-taking. *Psychon. Bull. Rev.* 12 957–968. 10.3758/bf03206432 16615316

[B139] Wilson S. J. Wales R. J. Pattison P. (1997). The representation of tonality and meter in children aged 7 and 9. *J. Exp. Child Psychol.* 64 42–66. 10.1006/jecp.1996.2331 9126627

[B140] Winkler I. Háden G. P. Ladinig O. Sziller I. Honing H. (2009). Newborn infants detect the beat in music. *Proc. Natl. Acad. Sci. U.S.A.* 106 2468–2471. 10.1073/pnas.0809035106 19171894 PMC2631079

[B141] Wu X. Westanmo A. Zhou L. Pan J. (2013). Serial binary interval ratios improve rhythm reproduction. *Front. Psychol.* 4:512. 10.3389/fpsyg.2013.00512 23964258 PMC3734359

[B142] Yu L. Myowa M. (2021). The early development of tempo adjustment and synchronization during joint drumming: a study of 18- to 42-month-old children. *Infancy* 26 635–646. 10.1111/infa.12403 33915019

[B143] Zentner M. Eerola T. (2010). Rhythmic engagement with music in infancy. *Proc. Natl. Acad. Sci. U.S.A.* 107 5768–5773. 10.1073/pnas.1000121107 20231438 PMC2851927

[B144] Zhao T. C. Kuhl P. K. (2016). Musical intervention enhances infants’ neural processing of temporal structure in music and speech. *Proc. Natl. Acad. Sci. U.S.A.* 113 5212–5217. 10.1073/pnas.1603984113 27114512 PMC4868410

[B145] Zou J. Feng J. Xu T. Jin P. Luo C. Zhang J. (2019). Auditory and language contributions to neural encoding of speech features in noisy environments. *Neuroimage* 192 66–75. 10.1016/j.neuroimage.2019.02.047 30822469

